# Elucidating oxide-ion and proton transport in ionic conductors using machine learning potentials

**DOI:** 10.1038/s41524-025-01807-y

**Published:** 2025-11-05

**Authors:** Ying Zhou, Sacha Fop, Abbie C. Mclaughlin, James A. Dawson

**Affiliations:** 1 Chemistry - School of Natural and Environmental Sciences, Newcastle University, Newcastle upon Tyne, UK; 2 Advanced Centre for Energy and Sustainability (ACES), The Chemistry Department, University of Aberdeen, Aberdeen, UK

**Keywords:** Materials for energy and catalysis, Materials science, Atomistic models

## Abstract

The design and understanding of oxide-ion and proton transport in solid electrolytes are pivotal to the development of fuel cells that can operate at reduced temperatures of <600 ^∘^C. Atomistic modelling and machine learning are playing ever more crucial roles in achieving this objective. In this study, using passive and active learning techniques, we develop moment tensor potentials (MTPs) for two promising ionic conductors, namely, Ba_7_Nb_4_MoO_20_ and Sr_3_V_2_O_8_. Our MTPs accurately reproduce ab initio molecular dynamics data and demonstrate strong agreement with density functional theory calculations for forces, energies and stresses. They successfully predict diffusion coefficients and conductivities for both oxide ions and protons, showing excellent agreement with experimental data and ab initio molecular dynamics results. Additionally, the MTPs accurately estimate migration barriers, thereby underscoring their robustness and transferability. Our findings highlight the potential of MTPs in significantly reducing computational costs while maintaining high accuracy, making them invaluable for simulating complex ion transport mechanisms and supporting the development of next-generation solid oxide fuel cells.

## Introduction

Fuel cells are a critical technology in the pursuit of sustainable energy solutions, offering high efficiency and low emissions^1–4^. Among the various types of fuel cells, solid oxide fuel cells (SOFCs) are notable for their robustness and large power outputs^2,5,6^. The development and optimization of new SOFCs that operate at lower temperatures are therefore crucial for achieving ambitious net-zero targets^7,8^. Understanding both oxide-ion and proton transport mechanisms is essential for optimizing the performance of mixed ionic conductors used in SOFC electrolytes and electrodes. Previous research has highlighted both Ba_7_Nb_4_MoO_20_ and Sr_3_V_2_O_8_ as promising solid electrolyte materials, with Ba_7_Nb_4_MoO_20_ exhibiting particularly high oxide-ion and proton conductivity^9–11^.

**Fig. 1:**
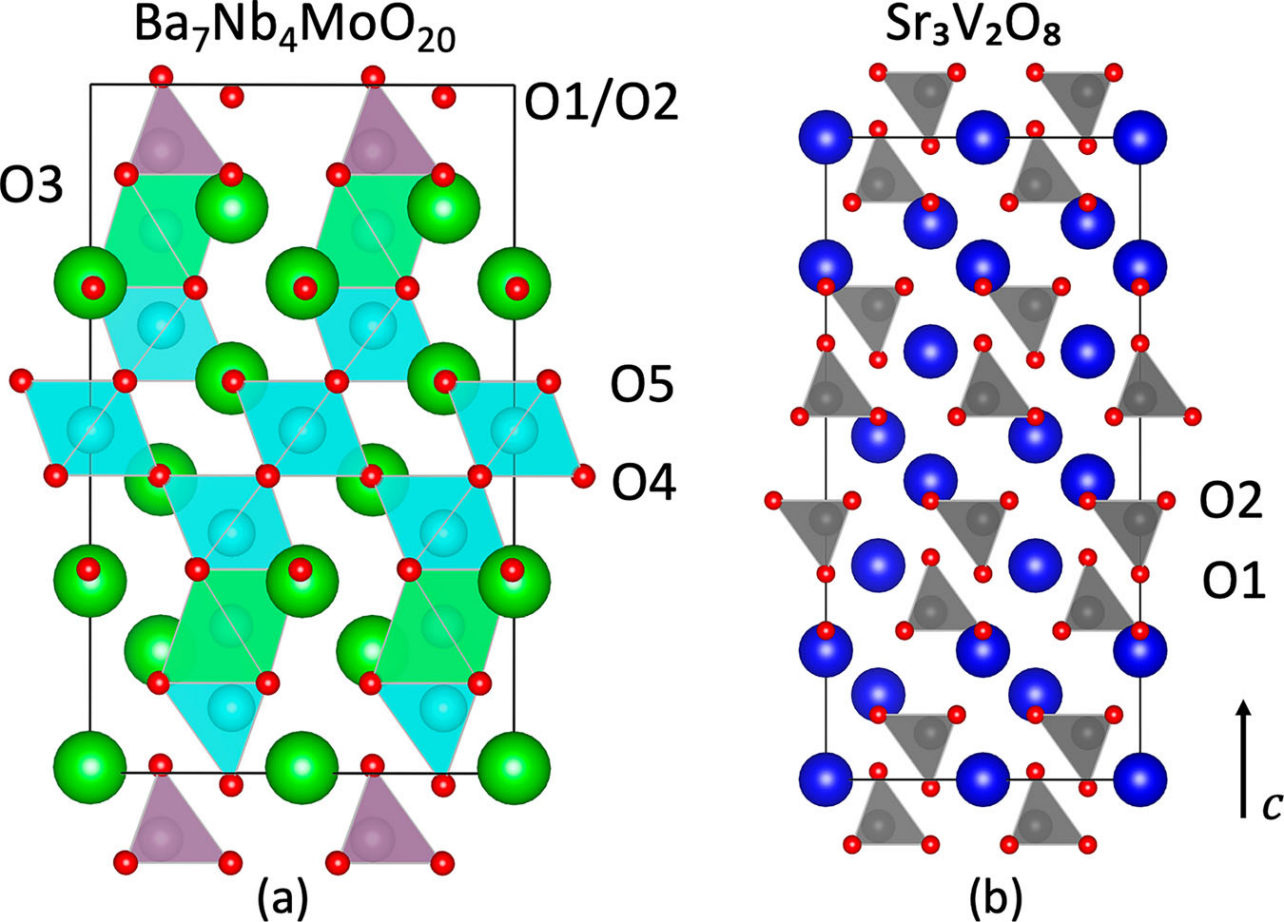
Crystal structures of Ba_7_Nb_4_MoO_20_ and Sr_3_V_2_O_8_. **a** Structure of Ba_7_Nb_4_MoO_20_ with partial O1 and O2 site occupancy causing average tetrahedral and octahedral coordination. **b** Structure of Sr_3_V_2_O_8_. Colours: green for Ba, light blue for Nb, pink for Mo, blue for Sr, grey for V, and red for O.

In addition to its excellent ionic conductivity, Ba_7_Nb_4_MoO_20_ is characterized by its redox stability, which is another crucial attribute for the efficient operation of SOFCs^10,12^. It has a complex hexagonal perovskite structure, consisting of palmierite Ba_3_M_2_O_8_ (M = Nb or Mo) and 12*R* perovskite Ba_7_Nb_4_MoO_20_ units^13,14^. Figure 1 illustrates the crystal structures and unique oxygen sites of Ba_7_Nb_4_MoO_20_ and Sr_3_V_2_O_8_. Ab initio molecular dynamics (AIMD) simulations of Ba_7_Nb_4_MoO_20_ have provided insights into the oxide-ion diffusion at high temperature (1100 K), indicating that diffusion primarily occurs via an interstitialcy mechanism involving the *a*–*b* plane through O1 and O2 sites along the palmierite-like layers, with additional contributions from O3–O1 exchange perpendicular to this plane^13^. Protons diffuse along palmierite-like layers in this material via low-energy pathways, aided by MO_x_ unit flexibility^9^.

Sr_3_V_2_O_8_ has also attracted interest as a result of its promising ionic conductivity^15–17^. The structural and electrical properties of Sr_3_V_2_O_8_ have been characterized using techniques such as X-ray and neutron diffraction, scanning electron microscopy and impedance spectroscopy. Bond-valence site energy calculations have explored its ionic transport mechanisms, revealing favourable migration barriers for ion transport^18^. Oxide-ion transport in Sr_3_V_2_O_8_ is known to occur via a vacancy mechanism. Additionally, AIMD simulations have explored proton diffusion, finding that the diffusion values are comparable to those of other high-performance proton conductors. The protons follow a 3D network with rotational hopping, occasionally trapped by cation vacancies. These computational findings are also supported by experimental measurements^19,20^.

Despite the insights from AIMD simulations, current methods for studying these materials are limited by high computational costs and challenges in accurately modelling large systems. This creates a gap between simulations and experimental results. Classical molecular dynamics simulations using machine learning potentials can help bridge this gap. Compared to empirical potentials, machine learning potentials provide higher accuracy while maintaining efficiency, enabling the simulation of larger systems over longer timescales^21–23^. These potentials are also scalable, allowing them to be applied to large systems after training on smaller datasets, and are transferable across different conditions and materials. The moment tensor potential (MTP), a type of machine learning potential, has been widely discussed and successfully applied to various chemical systems^24–26^. MTPs have been used to model alloys^27,28^, gas-phase reactions^29^, cathode coating materials^30^, and ionic conductors^31–37^.

In this work, we develop new MTPs for Ba_7_Nb_4_MoO_20_ and Sr_3_V_2_O_8_ to simulate their structural, mechanical and ion transport properties. Our MTPs can accurately capture interatomic interactions while significantly reducing computational costs. We apply these MTPs to conduct molecular dynamics simulations over extended timescales and on large systems. This approach allows us to study the dynamic behaviour and properties of these materials with unprecedented precision and efficiency. As a result, we are able to obtain important insights into their potential applications and performance.

## Results

### Validation of MTPs

Several properties were examined to validate the performance of the fitted MTPs. Table 1 shows the fitting errors relative to DFT. For each configuration, we calculate the difference between MTP and DFT values for energies, forces (per atom and Cartesian component), and stresses, then summarise over the dataset using MAE (maximal absolute error), RMSE (root mean square error) and AAD (average absolute error). For Sr_3_V_2_O_8_ ⋅ 0.33H_2_O, the errors in energy per atom are below the benchmark of 3 meV/atom, demonstrating a high level of accuracy. In terms of force errors, both materials show accurate force predictions, offering reliable insights into their behaviour.

**Table 1:** Errors in energies, atomic forces and stresses of the fitting dataset for each MTP with respect to the DFT values

	Ba_7_Nb_4_MoO_20_ ⋅ 0.5H_2_O			Sr_3_V_2_O_8_ ⋅ 0.33H_2_O		
	MAE	AAD	RMS	MAE	AAD	RMS
Errors in energy per atom (meV/atom)	11.1	2.9	3.6	9.1	2.1	2.7
Errors in forces (eV/Å)	5.52	0.33	0.39	1.71	0.25	0.29
Errors in stresses (kBar)	2.19	0.79	0.91	1.85	0.54	0.62

**Fig. 2:**
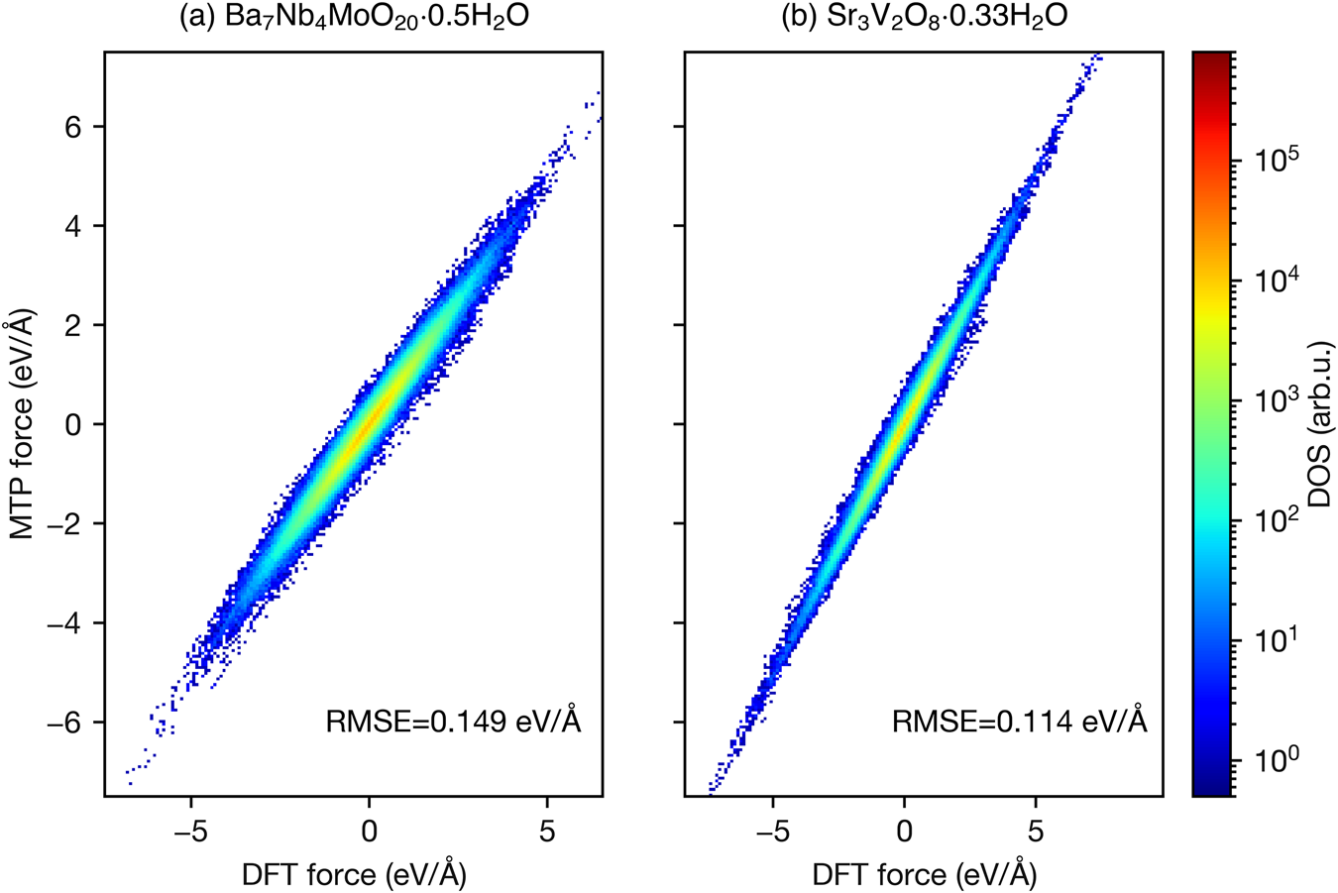
Atomic force correlations between MTPs and DFT. Density plot of force correlations for **a** Ba_7_Nb_4_MoO_20_ ⋅ 0.5H_2_O and **b** Sr_3_V_2_O_8_ ⋅ 0.33H_2_O, with colour gradient representing density of states (DOS).

To gain insight into the fitting performance, 1000 configurations from AIMD at 1200 K were selected. The force correlation between DFT and the MTPs is shown in Fig. 2. Each atom has three force components, resulting in a total of 804,000 and 504,000 force components for Ba_7_Nb_4_MoO_20_ and Sr_3_V_2_O_8_, respectively. The data points indicate a strong correlation between the two sets of force values, suggesting that the MTPs accurately predict forces similarly to DFT. The points are coloured based on density, with a gradient from blue (low density) to red (high density), revealing high-density areas along the diagonal. The RMSEs between the MTP and DFT force predictions are 0.149 eV/Å for Ba_7_Nb_4_MoO_20_ and 0.114 eV/Å for Sr_3_V_2_O_8_, which are well within the expected range for machine-learned potentials applied to complex oxides. These values are comparable to those reported in recent benchmarking studies (e.g., 0.09–0.27 eV/Å across different systems^38,39^) and reflect the high accuracy of the MTP models in capturing interatomic forces. The slightly lower RMSE observed for the Sr_3_V_2_O_8_ system can be attributed to its lower chemical complexity, containing four elements compared to five in Ba_7_Nb_4_MoO_20_. The increased number of chemical species in the Ba-based system introduces a broader range of local environments and bonding interactions, making the potential energy surface more complex and challenging to fit with the same level of accuracy.

Figure S1 shows the distribution of force errors, which are centred around zero, with most errors falling within a narrow range. This close agreement suggests that the MTP models effectively capture the force interactions predicted by DFT, providing a computationally efficient alternative without significant loss of accuracy. The comparison between the two materials shows a similar trend.

The lattice parameters and mechanical properties predicted by DFT and MTP simulations are listed in Table 2. The bulk modulus, shear modulus and Poisson’s ratio of Ba_7_Nb_4_MoO_20_ and Sr_3_V_2_O_8_ were calculated using second-order elastic constants derived from the strain-energy relationship using both DFT and the MTPs^40,41^.

**Table 2:** Comparison of DFT- and MTP-computed lattice parameters and mechanical properties of Ba_7_Nb_4_MoO_20_ and Sr_3_V_2_O_8_

	Ba_7_Nb_4_MoO_20_		Sr_3_V_2_O_8_	
	DFT	MTP	DFT	MTP
*a* (Å)	5.96	5.97	5.69	5.68
*c* (Å)	16.82	16.80	20.20	20.15
Bulk modulus (GPa)	80.74	79.14	75.94	83.28
Shear modulus (GPa)	36.42	38.44	33.06	31.60
Poisson’s ratio	0.31	0.29	0.31	0.33

For Ba_7_Nb_4_MoO_20_, the lattice parameters *a* and *c* are similar between DFT (5.96 and 16.82 Å) and the MTP (5.97 and 16.80 Å). The bulk and shear moduli exhibit small differences between the two methods. For Sr_3_V_2_O_8_, the lattice parameters *a* and *c* also show close agreement between DFT (5.69 and 20.20 Å) and the MTP (5.68 and 20.15 Å). The bulk and shear moduli differ slightly, with DFT values of 75.94 and 33.06 GPa, and MTP values of 83.28 and 31.60 GPa, respectively. The Poisson’s ratio shows a minor variation between both methods and materials. These differences are within acceptable accuracy limits for machine-learned potentials. Since the MTPs were trained on finite-temperature configurations rather than explicitly on 0 K elastic properties, the observed agreement is reasonable and sufficient for capturing the relevant structural and mechanical behaviour. This strong correlation with DFT calculations demonstrates the reliability and accuracy of the MTP models for predicting both structural and mechanical properties.

**Fig. 3:**
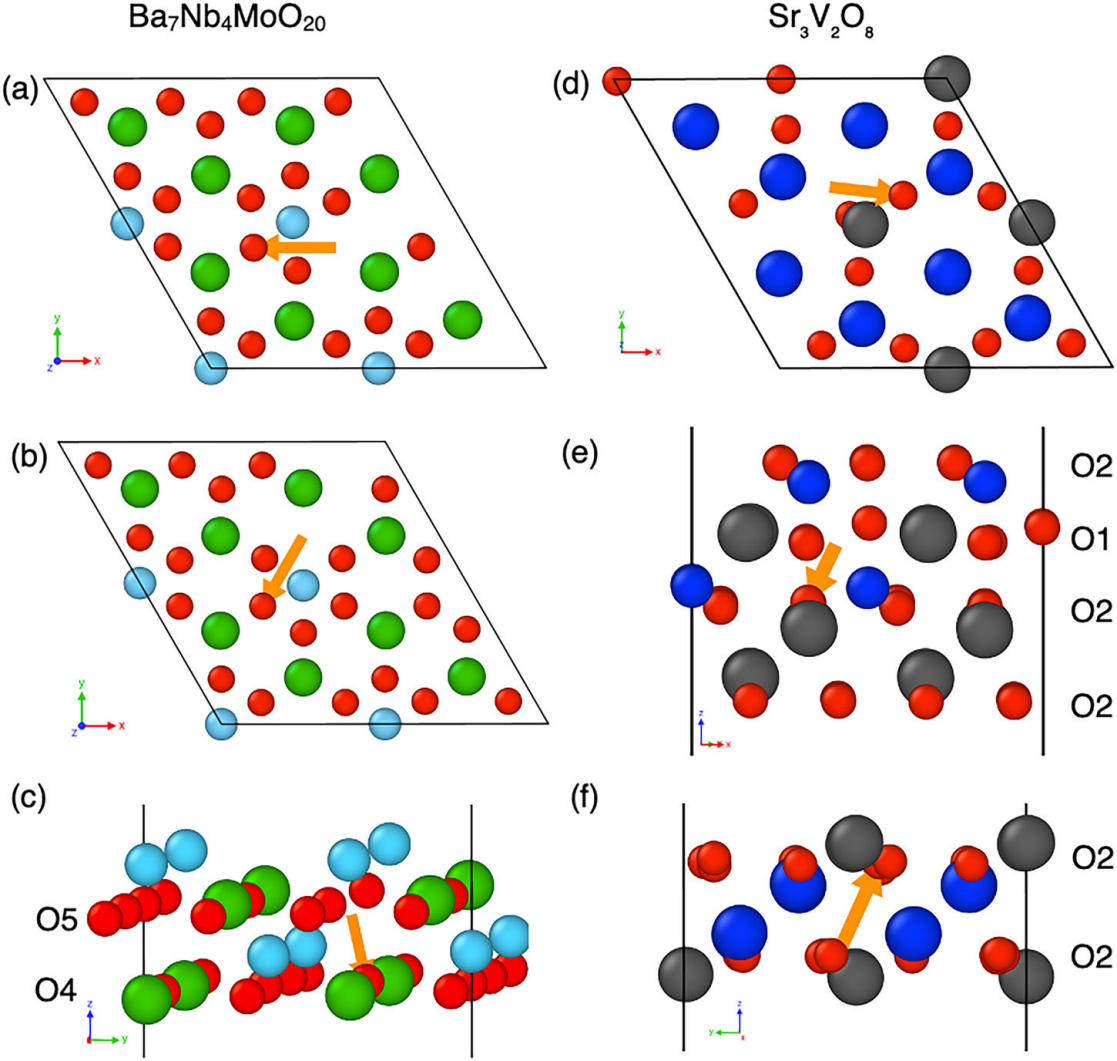
Oxide-ion migration pathways considered for Ba_7_Nb_4_MoO_20_ and Sr_3_V_2_O_8_. **a** O5–O5, 2.95 Å, **b** O5–O5, 2.95 Å, **c** O5–O4, 2.59 Å, **d** O2–O2, 2.53 Å, **e** O2–O1, 2.31 Å and **f** O2–O2, 2.79 Å. Orange arrows indicate the movement of oxide ions. Ba, Sr, Nb, V and O ions are represented by green, blue, light blue, grey and red spheres, respectively. Slices of the structures are shown, with other atoms omitted for clarity.

**Fig. 4:**
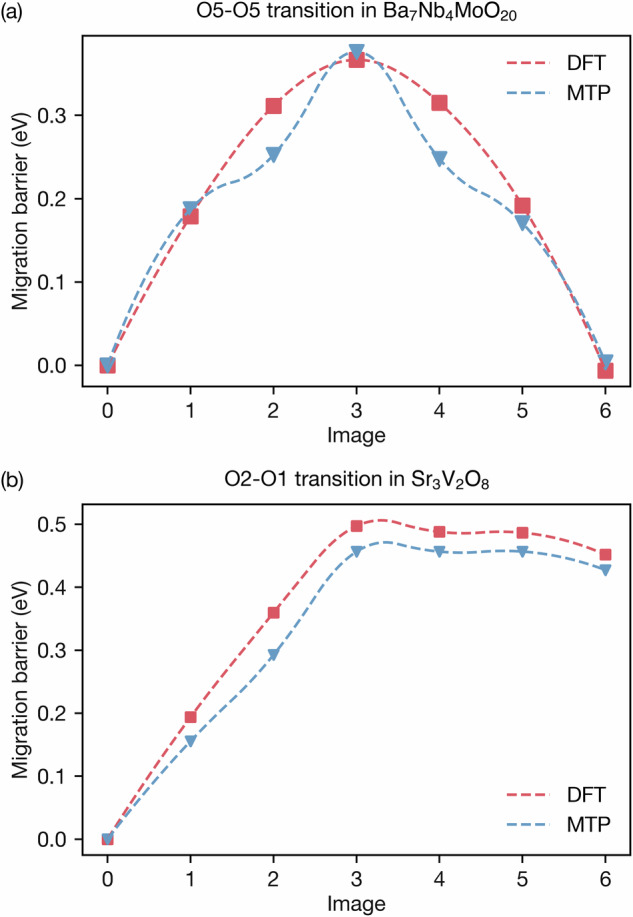
NEB energy curve for the single oxygen vacancy transition predicted by DFT (red dashed line) and MTP (blue dotted line) of each image. **a** Energy curve for the O5–O5 transition in Ba_7_Nb_4_MoO_20_ shown in Fig. 3b. **b** Energy curve for the O2–O1 transition in Sr_3_V_2_O_8_ shown in Fig. 3e.

The oxygen vacancy migration barriers were estimated using the nudged elastic band (NEB) method^42,43^. The climbing image method was enabled in VASP to achieve greater precision in determining these barriers. Five intermediate images were generated through linear interpolation between the two endpoints, with a spring constant of −5 eV/ Å^2^. Figure 3 presents the NEB calculations for three selected oxygen vacancy migration transitions in both Ba_7_Nb_4_MoO_20_ and Sr_3_V_2_O_8_. For each material, three distinct migration pathways were analysed to determine the energy barriers associated with the movement of oxygen vacancies. Figure 4a shows the energy curve of the O5–O5 transition Fig. 3b. In Fig. 4a, the *x*-axis represents the image index along the migration pathway, while the *y*-axis represents the vacancy migration barrier in eV. The DFT calculations (red dashed line) predict a barrier of 0.367 eV, while the MTP model (blue dotted line) shows a barrier of 0.376 eV. The MTP energy profile closely follows that of DFT, with only minor deviations, particularly small deviations near the transition region. These deviations are likely since some intermediate configurations along the path were not included in the MTP training data. As these points correspond to metastable, non-equilibrium states rather than true local minima, they do not affect the overall height or position of the energy barrier. This close alignment indicates that the MTP model can accurately replicate the migration barriers obtained from DFT, suggesting it is suitable for use in molecular dynamics simulations to predict oxide-ion diffusion accurately.

The NEB barriers, as predicted by DFT and the MTP models, are displayed in Table S1 for each transition. The table shows that the energy barriers for the transitions predicted by the MTP closely align with those calculated by VASP, indicating that the MTP model effectively captures the key features of the migration process.

### Oxide-ion and proton transport

**Fig. 5:**
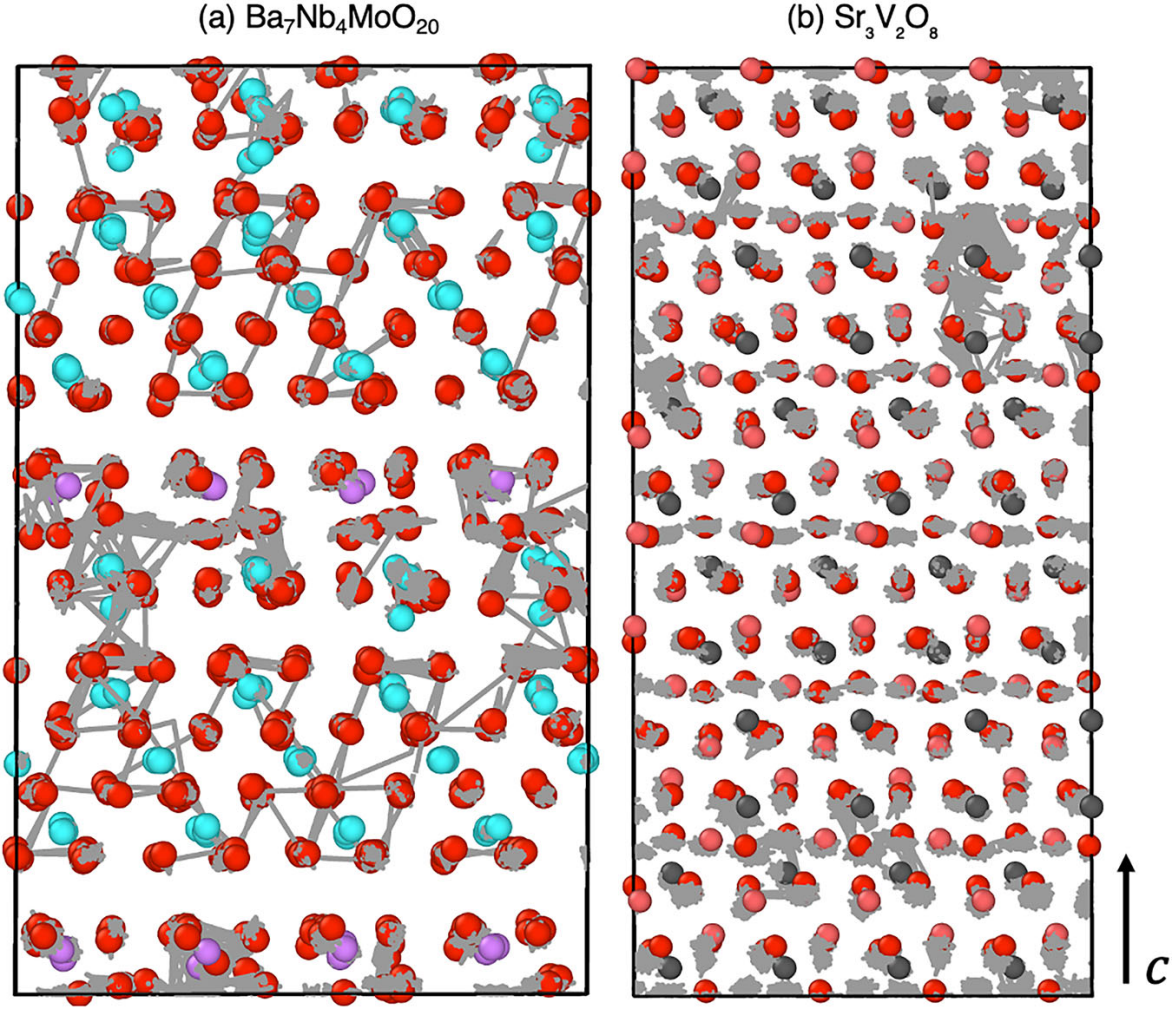
MTP-based molecular dynamics trajectories of oxide-ion diffusion. Oxide-ion diffusion trajectory in **a** Ba_7_Nb_4_MoO_20_ and **b** Sr_3_V_2_O_8_. Oxide ions are represented by red spheres. Ba and Sr ions are not shown for clarity.

Figure 5 shows the calculated trajectories of oxide-ion diffusion for Ba_7_Nb_4_MoO_20_ and Sr_3_V_2_O_8_. The trajectories were collected at 600 K over a simulation duration of 10 ns, following a 0.2 ns heating period. In Ba_7_Nb_4_MoO_20_, oxygen ion diffusion mainly occurs within the *a*–*b* plane through partially occupied oxygen sites. Molecular dynamics simulations showed a continuous two-dimensional diffusion pathway involving O1 and O2 sites. The flexibility of the MO_x_ polyhedra within the palmierite-like layers supports this process by allowing the reorganization of oxygen coordinations. Additionally, out-of-plane diffusion involving O3 oxygen atoms contributes to long-range oxygen ion transport^9,10,13,14^. The trajectories in Fig. 5b reveal that the movement of oxygen ions primarily involves O1 and O2 types. To quantitatively assess the anisotropy of oxygen transport, we calculated mean squared displacements (MSDs) resolved along the crystallographic *a*−*b* plane and *c* axis, as shown in Fig. S2 in the Supporting Information. In Ba_7_Nb_4_MoO_20_, the MSDs confirm that oxygen diffusion is highly anisotropic. Specifically, the in-plane (*a*−*b*) MSD is 4 times larger than the out-of-plane (*c*-axis) MSD throughout the simulation duration. This result quantitatively supports the notion of dominant two-dimensional transport within the palmierite-like layers, involving the O1/O2 partially occupied sites and the diffusion of O5 atoms. The smaller *c*-axis MSD indicates auxiliary transport involving O5–O4 and O3 sites, which contribute to long-range connectivity between layers.

In Sr_3_V_2_O_8_, diffusion occurs through O1–O1, O2–O2 and O1–O2 pathways. NEB calculations reveal a relatively high migration barrier of 1.24 eV for the O1-O1 hop, suggesting this pathway contributes less significantly to long-range transport. In contrast, the O1–O2 pathway shows a much lower barrier of 0.50–0.82 eV and the O2–O2 hops: both within the same layer (0.45 eV) and between layers (0.50 eV) also exhibit moderate barriers. These values indicate that oxygen diffusion is predominantly mediated by the O1–O2 and O2–O2 hops. These transitions form a more isotropic and interconnected three-dimensional network. This interpretation is consistent with the direction-resolved MSDs (Fig. S2). These observations are consistent with previous simulation results^18^. The O1–O1 and O2–O2 pathways involve oxygen ion hopping between identical oxygen sites within the tetrahedral framework, facilitated by the rotational dynamics of VO_4_ units. The O1–O2 pathway provides additional connectivity, enabling oxygen migration between different oxygen sites.

**Fig. 6:**
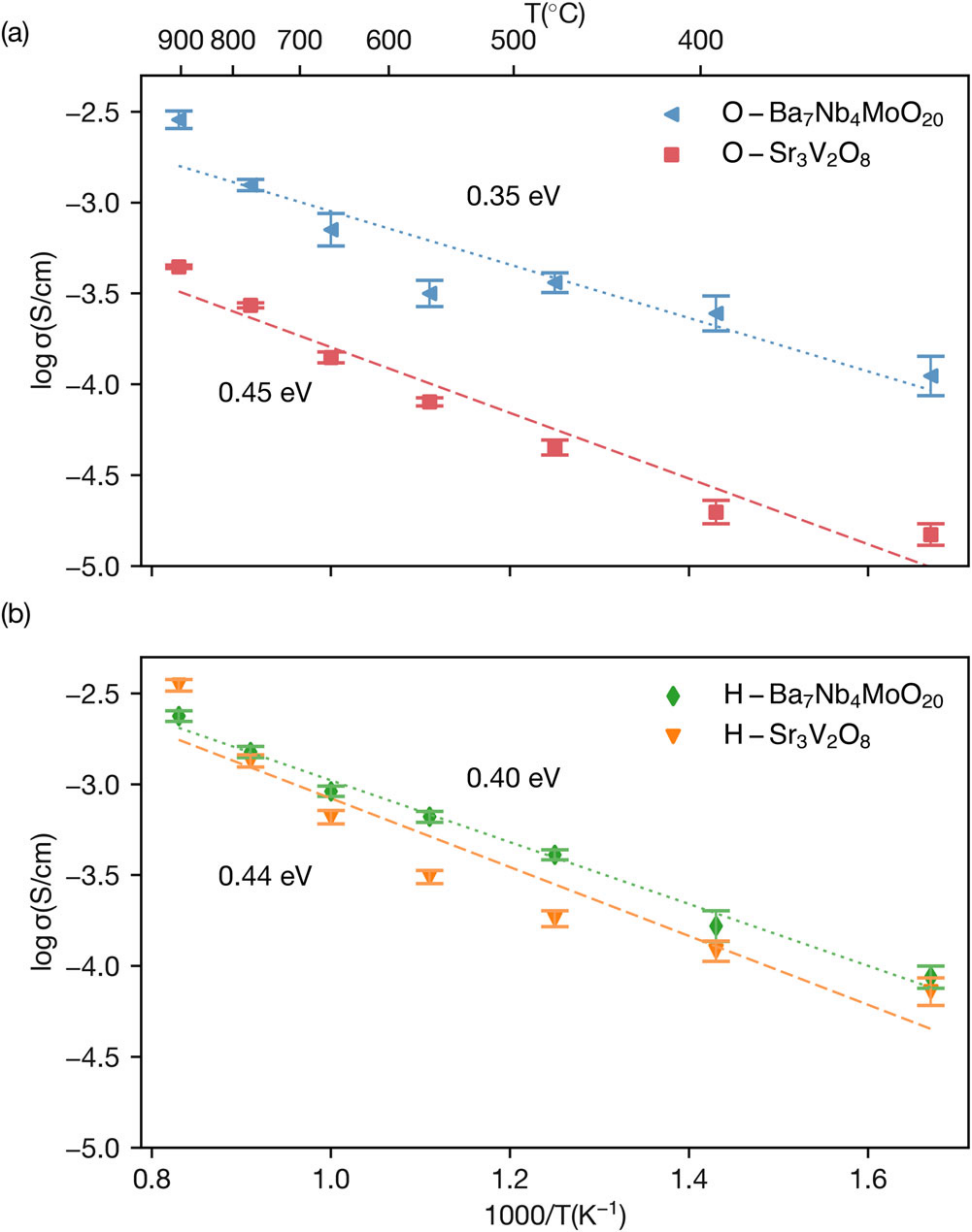
Arrhenius plots of oxide-ion and proton conductivity for Ba_7_Nb_4_MoO_20_ and Sr_3_V_2_O_8_. **a** Oxide-ion and **b** proton conductivity as a function of temperature for both materials.

Figure 6 provides a detailed comparison of the conductivity of Ba_7_Nb_4_MoO_20_ and Sr_3_V_2_O_8_ for both oxide ions and protons, based on temperature-dependent Arrhenius plots. The top plot illustrates the conductivity of oxide ions, where Ba_7_Nb_4_MoO_20_ shows a lower activation energy of 0.35 eV while Sr_3_V_2_O_8_ exhibits an activation energy of 0.45 eV, as indicated by the slope of the linear fit. This trend demonstrates the differences in the ionic conduction mechanisms of the two materials, with Ba_7_Nb_4_MoO_20_ showing relatively higher conductivity at lower temperatures due to its lower activation energy. Experimentally, the activation energies are measured at 0.30 eV for Ba_7_Nb_4_MoO_20_^9^ and 0.55 eV for Sr_3_V_2_O_8_^18^. A comparison of activation energies derived from MD, NEB, and experimental approaches is provided in Table S2 of the Supporting Information.

The bottom plot of Fig. 6 highlights the proton conductivity behaviour of the hydrated forms of the two materials. Ba_7_Nb_4_MoO_20_ shows an activation energy of 0.40 eV, maintaining its superior transport properties resulting from its different transport mechanisms compared to Sr_3_V_2_O_8_. For Sr_3_V_2_O_8_, the activation energy for proton conduction is slightly reduced to 0.44 eV compared to the value for oxide-ion conduction. These activation energy differences align with the observed trends in conductivity, where Ba_7_Nb_4_MoO_20_ consistently outperforms Sr_3_V_2_O_8_ in terms of proton transport under comparable conditions.

Previous AIMD simulations have provided insight into the diffusion behaviour of protons in these materials. For Ba_7_Nb_4_MoO_20_ ⋅ 0.5H_2_O, AIMD simulations predict a diffusion coefficient of 1.82 × 10^−5^ cm^2^/s at 1000 K, which is significantly higher than the value obtained from our molecular dynamics results, 3.58 × 10^−6^ cm^2^/s^9^. This indicates a more pronounced correction in Ba_7_Nb_4_MoO_20_, emphasizing the importance of revisiting the proton dynamics in these systems. For Sr_3_V_2_O_8_ ⋅ 0.33H_2_O, a proton diffusion coefficient of 3.43 × 10^−6^ cm^2^/s was reported at 800 K. However, our molecular dynamics simulations yield a slightly lower diffusion coefficient of 2.32 × 10^−6^ cm^2^/s^18^, suggesting a marginal underestimation in the earlier simulations.

When comparing the conductivity of protons, the results highlight the superior performance of Ba_7_Nb_4_MoO_20_. Experimental measurements show that it achieves a significantly higher proton conductivity of 4.0 × 10^−3^ S/cm at 500 ^∘^C^9^. In contrast, previous AIMD simulations predicted a proton conductivity of 8.04 × 10^−4^ S/cm for Sr_3_V_2_O_8_ at 800 K^18^, reflecting its moderate proton transport capability. This stark difference emphasizes the advantages of Ba_7_Nb_4_MoO_20_, which are likely due to its structural characteristics, higher proton mobility, and more favourable conduction pathways compared to Sr_3_V_2_O_8_. A summary of these diffusion and conductivity values is provided in Table S3 in the Supplementary Information.

Overall, the figure and accompanying data emphasise the role of structural and compositional factors in determining the ionic and protonic transport properties of these materials. Ba_7_Nb_4_MoO_20_ consistently exhibits lower activation energies and higher conductivity for both oxide ions and protons, which underscores its potential as a superior ion-conducting material. This enhanced transport behaviour can be attributed to its layered palmierite-perovskite structure, which incorporates disordered cation and oxygen sublattices, along with partially occupied oxygen sites (O1 and O2) that form a network of interlinked tetrahedral and octahedral units. These features provide a highly flexible and dynamically disordered environment that supports both oxide-ion and proton migration through continuous, low-barrier pathways. The role of structural disorder, flexible coordination environments, and polyhedral connectivity in enabling high ionic conductivity has been demonstrated in similar systems, such as Ba_3_NbMoO_8.5_ and related palmierite-type oxides^10,44,45^. Meanwhile, the findings also demonstrate the utility of combining molecular dynamics simulations and experimental data to evaluate and validate transport properties across different materials comprehensively.

## Discussion

Machine-learned interatomic potentials were successfully applied to model the structural and dynamic properties of Ba_7_Nb_4_MoO_20_ and Sr_3_V_2_O_8_. Using both passive and active learning approaches, we developed MTPs that achieve high fidelity with DFT reference data, accurately reproducing energies, forces and migration barriers. This validates the robustness and reliability in capturing key atomic-scale interactions, even without explicitly fitting to transition-state data. Additionally, the MTPs accurately captured the diffusion processes of oxide ions and protons in these materials, with calculated diffusion coefficients and activation energies that were in good agreement with both experimental and ab initio data. This close alignment confirms the ability of MTPs to accurately simulate ionic conductivity under various conditions, including the enhanced diffusion seen in hydrated environments.

Moreover, the computational efficiency of MTPs enables molecular dynamics simulations over nanosecond timescales and with systems comprising thousands of atoms, orders of magnitude beyond what is feasible with AIMD. It allows us to access long-range transport processes across a wide temperature range, significantly improving the reliability of the mean squared displacement data and enabling more accurate extraction of diffusion coefficients and ionic conductivities.

Overall, this work highlights the potential of MTPs as powerful tools for large-scale simulations of complex oxide materials. By greatly reducing the computational cost compared to traditional DFT methods, MTPs facilitate the exploration of larger systems and longer timescales, which are crucial for the development of next-generation solid electrolytes. These findings open the door to wider applications of machine learning techniques in materials science, particularly in the design and optimisation of materials for energy technologies.

## Methods

### Density functional theory simulations

Density functional theory (DFT) calculations were carried out using VASP with the projector augmented wave (PAW) method^46–49^ and the Perdew-Burke-Ernzerhof (PBE) exchange-correlation functional^50,51^. 2 × 2 × 1 supercells of Ba_7_Nb_4_MoO_20_ (128 atoms) and Sr_3_V_2_O_8_ (156 atoms) were employed. To model diffusivity under both dry and wet conditions, AIMD simulations were conducted on hydrated Ba_7_Nb_4_MoO_20_ ⋅ 0.5H_2_O and Sr_3_V_2_O_8_ ⋅ 0.33H_2_O, respectively. A *Γ*-centred 1 × 1 × 1 *k*-point mesh was used with a plane wave energy cutoff of 450 eV. Temperature control was maintained using a Nose-Hoover thermostat^52^ with a time step of 1 fs. AIMD simulations were run in the NVT ensemble across three volumes (with volumetric strains of 0 and ±5%) at 300, 600, 900 and 1200 K for 10 ps.

### Moment tensor potentials

MTPs were fitted to energies, forces and stresses from AIMD simulations using the MLIP-3 package^24,26^. MTPs describe the local atomic environment of the *i*th atom by the so-called moment tensor descriptors given by

1 $${M}_{n,\nu }=\sum _{j}{f}_{n,i,j}\left({r}_{ij}\right)\underbrace{{{\bf{r}}}_{ij}\otimes {{\bf{r}}}_{ij}\otimes \ldots \otimes {{\bf{r}}}_{ij}}_{\nu\, {\rm{times}}},$$

where the index *j* goes through all the neighbours of atom *i*. The symbol “⊗” stands for the outer product of vectors; therefore, **r**_*ij*_ ⊗ **r**_*ij*_ ⊗ … ⊗ **r**_*ij*_ is the tensor of rank *n* (*n* = 0, 1, …). The radial functions ${f}_{n,i,j}({r}_{ij})$ are a set of polynomial basis functions defined over the interatomic distance, multiplied by a smooth cutoff function to ensure locality. These functions form the radial part of the moment tensor descriptors and are used to capture how atomic interactions vary with distance. Their number and form are determined by the choice of radial basis size and cutoff radius *R*_cut_. Different tensor contractions of these moments *M* form basis functions of the MTP. A linear combination of these basis functions is parameterised to reproduce the energies, atomic forces and stresses from AIMD runs.

**Fig. 7:**
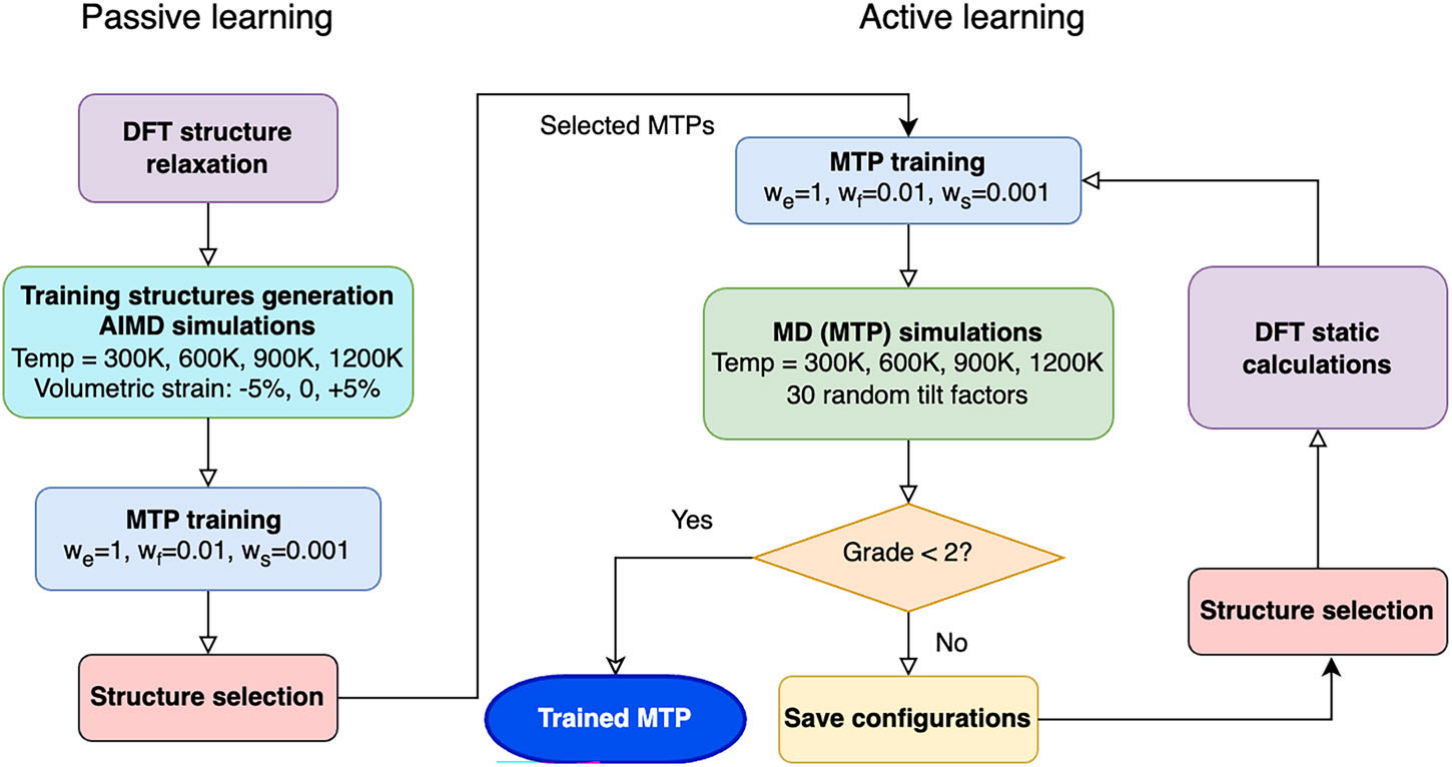
Passive and active learning approaches for MTP development. The left part shows passive learning using DFT and AIMD data for initial MTP training, while the right part illustrates active learning.

Figure 7 illustrates the flowchart of passive and active learning procedures used in our fitting process. All fittings employed a level-16 MTP, which includes 2068 fitting parameters for Ba_7_Nb_4_MoO_20_ ⋅ 0.5H_2_O and 1636 fitting parameters for Sr_3_V_2_O_8_ ⋅ 0.33H_2_O^25^. The fitting weights for the energies, atomic forces and stresses were set to 1, 0.01 Å^2^ and 0.001 Å^6^, respectively. Initially, 300 AIMD snapshots were collected at intervals of 0.1 ps from each temperature, resulting in a total of 1200 configurations used at the start of passive training. We then performed three rounds of passive learning, during which additional configurations were incrementally selected from the full AIMD datasets based on the model uncertainty. Ultimately, 2551 configurations of Ba_7_Nb_4_MoO_20_ ⋅ 0.5H_2_O and 2116 configurations of Sr_3_V_2_O_8_ ⋅ 0.33H_2_O were utilized in the passive learning procedure.

The active learning process for fitting MTPs began with the training of MTPs on a large set of configurations. Following this, molecular dynamics simulations were conducted using the refined MTPs at a range of temperatures (300, 600, 900 and 1200 K). These simulations were designed to sample a diverse range of atomic configurations by performing 30 independent MD runs with different random initial velocities. After running the simulations, the accuracy and reliability of the MTPs were evaluated using the extrapolation grade *γ*^26,53^. This metric quantifies how far a given atomic configuration lies from the training domain. Configurations with typically *γ* ⩽ 2 are considered to fall within the model’s interpolation regime and are thus reliably predicted. If *γ* exceeds this threshold, the configuration is flagged as extrapolative and added to a queue for DFT recalculation. These new DFT results are then incorporated into the training set in subsequent iterations. The active learning loop continues until all sampled configurations fall below the *γ* ⩽ 2 threshold, ensuring that the final potential is both accurate and robust across the explored configuration space. By iteratively refining the MTPs through active learning, the training dataset is enriched with previously unseen configurations, thereby improving the robustness and transferability of the resulting potential across a broad range of relevant atomic environments.

### Molecular dynamics simulations

All energy and force calculations with the MTPs were performed using the LAMMPS package^54,55^. Molecular dynamics simulations were initially performed in the NPT ensemble for 0.2 ns to allow volume relaxation, followed by 10 ns simulations in the NVT ensemble at temperatures ranging from 600 to 1200 K in 100 K intervals. These simulations employed an accurate MTP on supercells of 4 × 4 × 2 for Ba_7_Nb_4_MoO_20_ (1024 atoms) and Sr_3_V_2_O_8_ (1248 atoms). Oxygen vacancies were introduced to both Sr_3_V_2_O_8_ and Ba_7_Nb_4_MoO_20_ at a concentration of 1.56% and 1.87%, respectively, to enable oxide-ion transport. Ten independent molecular dynamics runs were executed, and the mean square displacements (MSDs) were calculated and averaged to determine the diffusion coefficient (*D*_*i*_)^56^:

2 $${D}_{i}=\frac{1}{6}\mathop{\lim }\limits_{t\to \infty }\frac{d}{dt}\langle {[{\bf{r}}(t)-{\bf{r}}(0)]}^{2}\rangle$$

where **r**(*t*) and **r**(0) represent the positions at time *t* and the initial reference position, respectively. The Nernst-Einstein equation^57^ was used to calculate the ionic conductivity (*σ*) of the system:

3 $$\sigma =\frac{{e}^{2}}{{\kappa }_{B}TV}\mathop{\sum }\limits_{i}{N}_{i}{q}_{i}^{2}{D}_{i}$$

where *e* is the electric charge unit, *κ*_*B*_ is Boltzmann constant, *T* is temperature, *q*_*i*_ is the charge of ion of type *i* and *N*_*i*_ is the number of ions of type *i*.

## Supplementary information

Supplementary information

## Data Availability

The training dataset can be openly accessed at 10.6084/m9.figshare.29552567.v1.
