# Supplementary material for: Elucidating oxide-ion and proton transport in ionic conductors using machine learning potentials
Source: NPJ Comput Mater. 2025 Nov 5;11(1):328. doi: 10.1038/s41524-025-01807-y (PMC12589113; doi:10.1038/s41524-025-01807-y)
Supplement: Supplementary file 1 — Supplementary information [file 41524_2025_1807_MOESM1_ESM.pdf]

Supporting Information: Elucidating Oxide-Ion and Proton Transport in Ionic Conductors using Machine Learning Potentials

Ying Zhou,*[,1] Sacha Fop,[2] Abbie C. Mclaughlin,[2] and James A. Dawson*[,1]

[1] *Chemistry – School of Natural and Environmental Sciences, Newcastle University, Newcastle upon Tyne, NE1 7RU, UK*

[2] *Advanced Centre for Energy and Sustainability (ACES), The Chemistry Department, University of Aberdeen, Aberdeen, AB24 3UE, UK*

* E-mail: ying.zhou@newcastle.ac.uk; james.dawson@newcastle.ac.uk

Supplementary Note 1: Summary of Supporting Data

The data presented in this Supporting Information file provide quantitative validation of the machine-learned Moment Tensor Potentials (MTPs) used to model oxide-ion and proton transport in $Ba_7Nb_4MoO_{20}$ and $Sr_3V_2O_8$.

Supplementary Figure S1 illustrates the distribution of force errors between MTP predictions and DFT reference values for the hydrated $Ba_7Nb_4MoO_{20}{\cdot}0.5H_2O$ and $Sr_3V_2O_8{\cdot}0.33H_2O$. The low error values confirm the fidelity of the trained potentials across relevant local environments.

Table S1 compares NEB-calculated migration barriers obtained from DFT and MTP for selected diffusion pathways in both materials. The close agreement between MTP and DFT barrier heights underscores the accuracy of the models in reproducing the energetics of transition states.

Supplementary Figure S2 shows the oxygen mean squared displacement (MSD) in the a-b plane and along the c axis at 1000 K. The MSD trends reveal anisotropic ionic mobility, which means that conduction is more restricted along the layered structure.

Table S2 reports activation energies for oxygen and proton conduction determined from MD, NEB (DFT and MTP), and experimental results. Notably, proton conduction in $Ba_7Nb_4MoO_{20}$ exhibits a lower activation energy (0.40 eV from MD) compared to $Sr_3V_2O_8$ (0.44 eV), consistent with the observed conductivity trends discussed in the main text.

Table S3 presents a comparison of proton diffusion coefficients and conductivities across different methods, including AIMD, classical MD, and experiments. These results demonstrate the capability of the trained MTPs to quantitatively capture diffusion behaviour over a wide range of temperatures and longer time scales.

In summary, the supplementary data collectively validate the use of MTPs for modelling complex ionic transport mechanisms in oxide materials. The consistency across methods reinforces the reliability of the potentials and supports the main conclusions of this study.

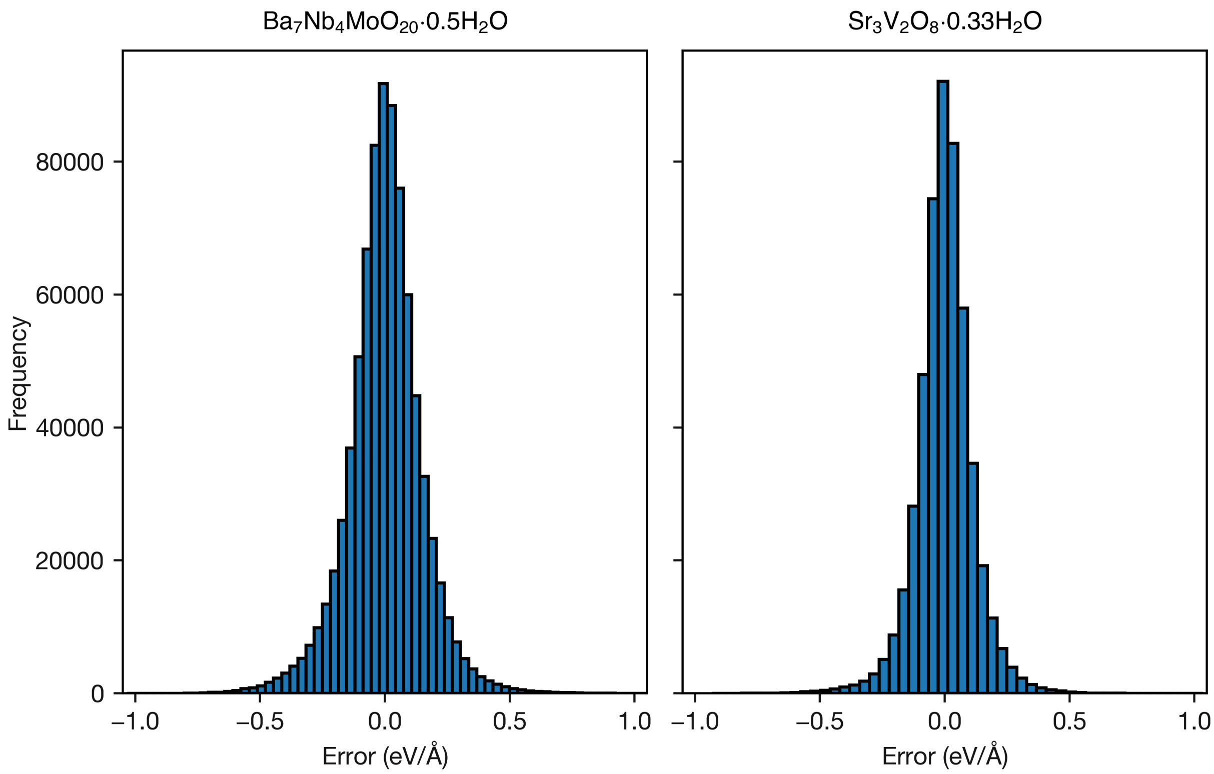

Figure S1: Distributions of force errors between MTP and DFT calculations for $Ba_7Nb_4MoO_{20}$·0.5H_2O and $Sr_3V_2O_8$·0.33H_2O.

Table S1: Comparison of DFT- and MTP-computed NEB barriers (eV) of $Ba_7Nb_4MoO_{20}$ and $Sr_3V_2O_8$ for transitions in Figure 3.

Material	Transition	DFT	MTP
$Ba_7Nb_4MoO_{20}$	a	0.32	0.37
	b	0.37	0.38
	c	0.34	0.38
$Sr_3V_2O_8$	d	0.41	0.45
	e	0.50	0.56
	f	0.51	0.48

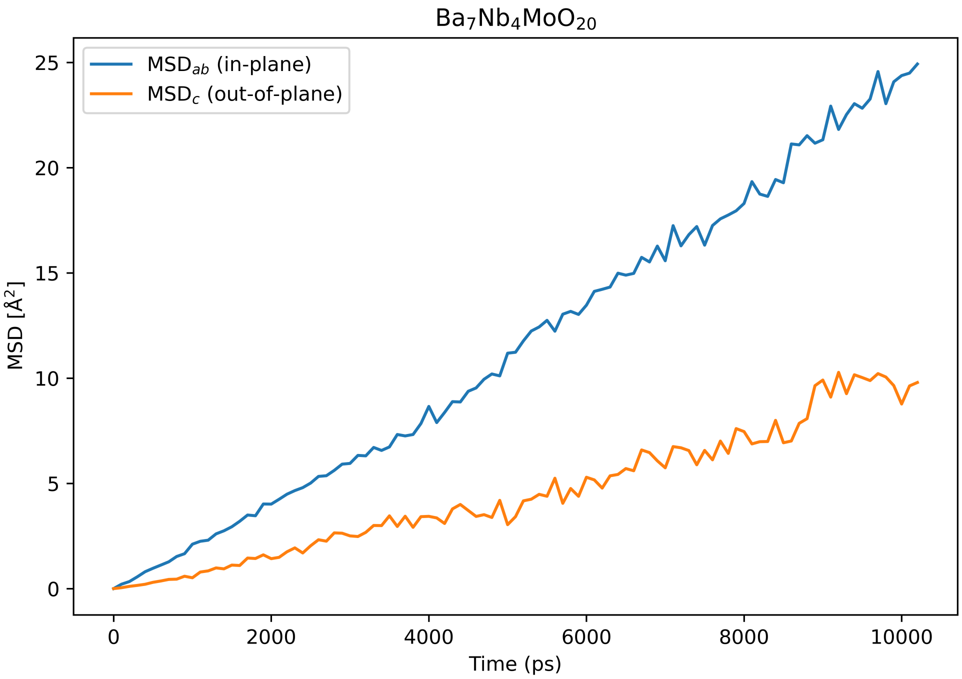

(a)

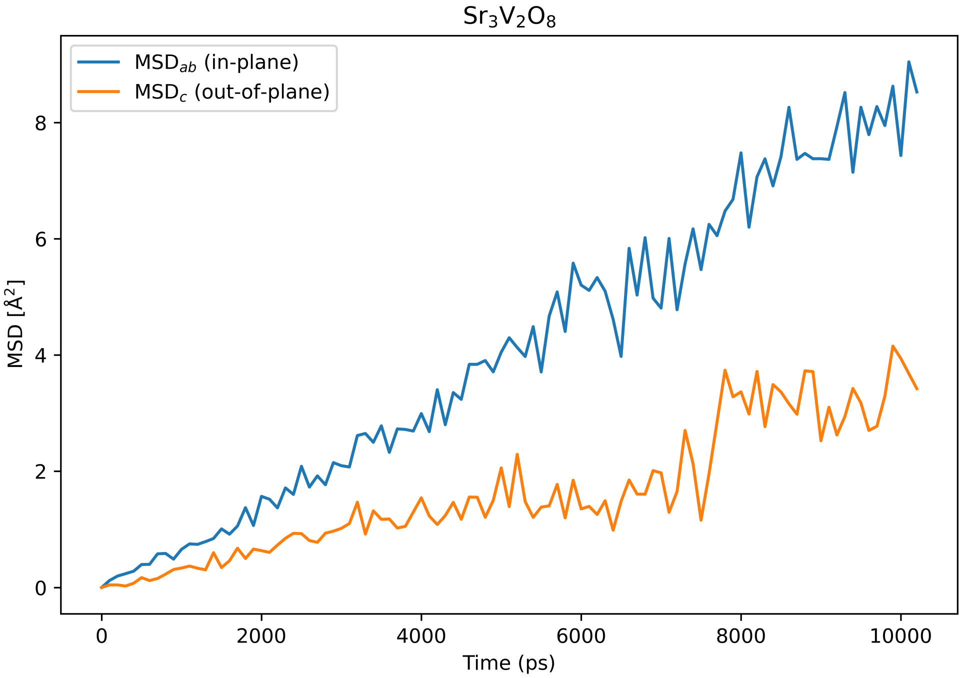

(b) msd

Figure S2: Oxygen mean squared displacement (MSD) in $Ba_7Nb_4MoO_{20}$ and $Sr_3V_2O_8$ at 1000 K. (a) Oxygen MSD in the *a*-*b* plane and along the *c* axis for $Ba_7Nb_4MoO_{20}$ at 1000 K. (b) Oxygen MSD in the *a*-*b* plane and along the *c* axis for $Sr_3V_2O_8$ at 1000 K. The anisotropic MSD profiles highlight differences in oxygen ion mobility within and between the structural layers of the two materials.

Table S2: Comparison of activation energy barriers (eV) between experiment, MD, and NEB methods for $Ba_7Nb_4MoO_{20}$ and $Sr_3V_2O_8$.

System		Method	Activation Energy (eV)	Notes
$Ba_7Nb_4MoO_{20}$	Oxygen	Experiment	0.30	Ref. [9]
		MD	0.35	This work
		NEB (DFT)	0.37	O5−O5 pathway in Fig.3(b)
		NEB (MTP)	0.38	O5−O5 pathway in Fig.3(b)
	Proton	Experiment	0.14-0.70	Ref. [9]
		MD	0.40	This work
$Sr_3V_2O_8$	Oxygen	Experiment	0.55	Ref. [18]
		MD	0.45	This work
		NEB (DFT)	0.50	O2−O2 pathway in Fig.3(e)
		NEB (MTP)	0.46	O2−O2 pathway in Fig.3(e)
	Proton	Experiment	-	-
		MD	0.44	This work

Table S3: Comparison of proton diffusion coefficients and conductivities for $Ba_7Nb_4MoO_{20}{\cdot}0.5H_2O$ and $Sr_3V_2O_8{\cdot}0.33H_2O$ from different methods.

Material	Method	Temperature	D_H (cm^2/s)	σ_H (S/cm)	Note
$Ba_7Nb_4MoO_{20}{\cdot}0.5H_2O$	AIMD	1000 K	1.82×10^{-5}	–	Ref. [9]
	MD	1000 K	3.58×10^{-6}	–	This work
	Experiment	773 K	–	4.0×10^{-3}	Ref. [9]
$Sr_3V_2O_8{\cdot}0.33H_2O$	AIMD	800 K	3.43×10^{-6}	8.04×10^{-4}	Ref. [18]
	MD	800 K	2.32×10^{-6}	–	This work
	Experiment	–	–	–	Not available
